# Supplementary material for: Marine-Derived Secondary Metabolites as Promising Epigenetic Bio-Compounds for Anticancer Therapy
Source: Mar Drugs. 2020 Dec 31;19(1):15. doi: 10.3390/md19010015 (PMC7824531; doi:10.3390/md19010015)
Supplement: Supplementary file 1 [file marinedrugs-19-00015-s001.pdf]

Table S1. Recent registered clinical trials on phenolic compounds with epigenetic mechanisms in different cancers alone and in co-treatment.

Drug	Study	Disease	Phase	Recruitment status	NCT number
Panobinostat	Oral Panobinostat (LBH589) with Chemotherapy in Patients < 65 Years Old	Acute Myeloid Leukemia	Phase 1	Completed	NCT01242774
Panobinostat	Access to Single Agent Panobinostat for Patients who are on s.a. Panobinostat Treatment in a Novartis-sponsored Study	Hematologic Neoplasms	Phase 2	Completed	NCT01802879
Panobinostat	Cisplatin and Pemetrexed in Combination with Panobinostat	Solid Tumors-Non-Small Cell Lung Cancer	Phase 1	Completed	NCT01336842
Panobinostat	Panobinostat and Lenalidomide	Adult Lymphocyte Depletion Hodgkin Lymphoma,<br>Adult Lymphocyte Predominant Hodgkin Lymphoma,<br>Adult Mixed Cellularity Hodgkin Lymphoma,<br>Adult Nodular Lymphocyte Predominant Hodgkin Lymphoma,<br>Adult Nodular Sclerosis Hodgkin Lymphoma,<br>Recurrent Adult Hodgkin Lymphoma	Phase 2	Completed	NCT01460940
Panobinostat	Panobinostat (LBH589) in Patients with Metastatic Melanoma	Melanoma,<br>Malignant Melanoma	Phase 1	Completed	NCT01065467
Panobinostat	Panobinostat Maintenance after HSCT for High-risk AML and MDS	Acute Myeloid Leukemia<br>Myelodysplastic Syndromes	Phase 3	Recruiting	NCT04326764
Panobinostat	Trial of Panobinostat in Children with Diffuse Intrinsic Pontine Glioma	Glioma	Phase 1	Recruiting	NCT02717455
Panobinostat	A Disease Registry Encompassing the Care of Patients with Multiple Myeloma on Panobinostat (RECOMM)	Multiple Myeloma		Recruiting	NCT04150289
Panobinostat	CINC424A2X01B Rollover Protocol	Primary Myelofibrosis,<br>Chronic Idiopathic Myelofibrosis	Phase 4	Recruiting	NCT02386800

Panobinostat	Panobinostat, Gemcitabine Hydrochloride, Busulfan, and Melphalan before Stem Cell Transplant in Treating Patients with Refractory or Relapsed Multiple Myeloma	Plasma Cell Leukemia, Plasmacytoma, Recurrent Plasma Cell Myeloma, Refractory Plasma Cell Myeloma	Phase 2	Recruiting	NCT02506959
Panobinostat	PDR001 in Combination with LCL161, Everolimus or Panobinostat	Colorectal Cancer, Non-small Cell Lung Carcinoma (Adenocarcinoma), Triple Negative Breast Cancer, Renal Cell Carcinoma	Phase 1	Recruiting	NCT02890069
Panobinostat	Infusion of Panobinostat (MTX110) into the Fourth Ventricle in Children and Adults with Recurrent Medulloblastoma	Medulloblastoma	Early Phase 1	Recruiting	NCT04315064
Panobinostat	Tolerability, Efficacy, and Safety Study of Pazopanib in Combination with PCI-24781 in Patients with Metastatic Solid Tumors	Metastatic Solid Tumors	Phase 1	Recruiting	NCT01543763
Panobinostat	CED of MTX110 Newly Diagnosed Diffuse Midline Gliomas	Diffuse Intrinsic Pontine Glioma, Diffuse Pontine and Thalamic Gliomas, Diffuse Midline Glioma	Phase 1	Recruiting	NCT04264143
Panobinostat	Serial Measurements of Molecular and Architectural Responses to Therapy (SMMART) PRIME Trial	Accelerated Phase Chronic Myelogenous Leukemia, BCR-ABL1 Positive, Anatomic Stage IV Breast Cancer AJCC v8, Hodgkin Lymphoma, Non-Hodgkin Lymphoma, Others	Phase 1	Recruiting	NCT03878524
Trichostatin A	Tolerability Study of Trichostatin A in Subjects with Relapsed or Refractory Hematologic Malignancies	Relapsed or Refractory Hematologic Malignancies	Phase 1	Recruiting	NCT03838926
Trichostatin A	EUS-guided Laser Ablation in Pancreatic Adenocarcinoma	Pancreatic Adenocarcinoma, Pancreas Cancer	Not applicable	Recruiting	NCT03784417

Vorinostat	Vorinostat in Combination with Chemotherapy in Relapsed/Refractory Solid Tumors and CNS Malignancies	Ewing Sarcoma,<br>Rhabdomyosarcoma,<br>Wilms Tumor	Phase 1	Recruiting	NCT04308330
Vorinostat	Effect of Vorinostat on ACTH Producing Pituitary Adenomas in Cushing s Disease	Cushing's Disease	Phase 2	Recruiting	NCT04339751
Vorinostat	Study to Assess Safety and Activity of Combination Therapy of VRC07-523LS and Vorinostat on HIV-infected Persons	HIV-1 Infection	Phase 1	Recruiting	NCT03803605
Vorinostat	Clinical Trial to Determine Tolerable Doses of Vorinostat in Patients with Mild Alzheimer’s Disease (VostatAD01)	Alzheimer’s Disease	Phase 1	Recruiting	NCT03056495
Vorinostat	Pembro and Vorinostat for Patients with Stage IV Non-small Cell Lung Cancer	Lung Cancer,<br>Non-small Cell Lung Cancer	Phase 1	Recruiting	NCT02638090
Vorinostat	Efficacy of Immunotherapy Plus a Drug in Patients with Progressive Advanced Mucosal Cancer of Different Locations	Squamous Cell Lung Cancer,<br>Vulvar Cancer,<br>Penile Cancer,<br>Head and Neck Squamous Cell Carcinoma,<br>Anal Cancer	Phase 2	Recruiting	NCT04357873
Vorinostat	Safety and Tolerability of Vorinostat for the Treatment of Moderate-to-Severe Crohn’s Disease	Crohn's Disease	Phase 1<br>Phase 2	Recruiting	NCT03167437
Vorinostat	Vorinostat Dose-escalation after Allogeneic Hematopoietic Cell Transplantation	Acute Myeloid Leukemia,<br>Myelodysplastic Syndromes,<br>Mixed Phenotype Acute Leukemia,<br>Juvenile Myelomonocytic Leukemia	Phase 1	Recruiting	NCT03843528
Vorinostat	Vorinostat for Graft vs Host Disease Prevention in Children, Adolescents and Young Adults Undergoing Allogeneic Blood and Marrow Transplantation	Hematologic Diseases<br>Acute Myeloid Leukemia in Remission,<br>Acute Lymphoblastic Leukemia in Remission,<br>Chronic Myelogenous Leukemia, Chronic Phase,	Phase 1<br>Phase 2	Recruiting	NCT03842696

	Chronic Myelogenous Leukemia, Accelerated Phase, Chronic Myelogenous Leukemia, Blastic Phase, Myelodysplastic Syndromes, Mantle Cell Lymphoma, Follicular Lymphoma, Diffuse Large B Cell Lymphoma, Non-Hodgkin Lymphoma	

Table S2. Recent registered clinical trials on cyclic peptides with epigenetic mechanisms in different cancers alone and in co-treatment.

Drug	Study	Disease	Phase	Recruitment status	NCT number
Romidepsin	Romidepsin Maintenance after Allogeneic Stem Cell Transplantation	Cutaneous T-cell Lymphoma, T-Prolymphocytic Leukemia, T-Large Granulocytic Leukemia, T-Lymphoblastic Leukemia/Lymphoma, Peripheral T-Cell Lymphoma	Phase 1	Recruiting	NCT02512497
Romidepsin	Pralatrexate + Romidepsin in Relapsed/Refractory Lymphoid Malignancies (PDX+Romi)	Lymphoid Malignancies, Multiple Myeloma, Lymphoma, Hodgkin Lymphoma, Non-Hodgkin Lymphoma	Phase 1/Phase 2	Recruiting	NCT01947140
Romidepsin	Romidepsin and Lenalidomide in Treating Patients with Previously Untreated Peripheral T-Cell Lymphoma	Adult Nasal Type Extranodal NK/T-cell Lymphoma, Anaplastic Large Cell Lymphoma, Angioimmunoblastic T-cell Lymphoma	Phase 2	Recruiting	NCT02232516
Romidepsin	A Study to Assess the Feasibility of Romidepsin Combined with Brentuximab Vedotin in Cutaneous T-cell Lymphoma	Cutaneous T-cell Lymphoma	Phase 1	Recruiting	NCT02616965
Romidepsin	ISTODAX® for Intravenous Infusion Drug Use Results Survey – Relapsed or Refractory Peripheral T-Cell Lymphoma	Lymphoma, T-Cell, Peripheral		Recruiting	NCT03742921
Romidepsin	Durvalumab in Different Combinations with Pralatrexate, Romidepsin and Oral 5-Azacitidine for Lymphoma	Lymphoma, T-Cell	Phase 1/2		NCT03161223
Romidepsin	Trial of Duvelisib in Combination with either Romidepsin or Bortezomib in Relapsed/Refractory T-cell Lymphomas	Relapsed/Refractory T-cell Lymphomas, Lymphoma	Phase 1	Recruiting	NCT02783625
Romidepsin	Determination Safety and Tolerability of Epigenetic and Immunomodulating Drugs in	Pancreas Cancer, Pancreatic Adenocarcinoma,	Phase 1/2	Recruiting	NCT04257448

	Combination with Chemotherapeutics in Patients Suffering from Advanced Pancreatic Cancer. (SEPION)	Pancreatic Ductal Adenocarcinoma			
Romidepsin	Efficacy and Safety of Oral Azacitidine (CC-486) Compared to Investigator's Choice Therapy in Patients with Relapsed or Refractory Angioimmunoblastic T Cell Lymphoma	Lymphoma, T-Cell	Phase 3	Recruiting	NCT03703375
Romidepsin	Efficacy and Safety of Oral Azacitidine Compared to Investigator's Choice Therapy in Patients with Relapsed or Refractory AITL	Relapsed Angioimmunoblastic T-Cell Lymphoma, Refractory Angioimmunoblastic T-cell Lymphoma	Phase 3	Recruiting	NCT03593018
Romidepsin	High Throughput Drug Sensitivity Assay and Genomics-Guided Treatment of Patients with Relapsed or Refractory Acute Leukemia	Recurrent Acute Leukemia of Ambiguous Lineage, Recurrent Acute Lymphoblastic Leukemia, Recurrent Acute Myeloid Leukemia	Not applicable	Recruiting	NCT02551718
Romidepsin	Long-term Use of Romidepsin in Patients with CTCL	Cutaneous T-cell Lymphoma		Completed	NCT02296398
Romidepsin	A Pilot Study of Romidepsin in Relapsed or Refractory Extranodal NK/T-cell Lymphoma (Ro-ENKTL)	Histologically Proven Extranodal NK/T-cell Lymphoma	Early phase 1	Completed	NCT01913119
Romidepsin	A Trial of Oral 5-azacitidine in Combination with Romidepsin in Advanced Solid Tumors, with an Expansion Cohort in Virally Mediated Cancers and Liposarcoma	Solid Tumors, Virally Mediated Cancers and Liposarcoma	Phase 1	Completed	NCT01537744
Romidepsin	Influence of Ketoconazole on the Pharmacokinetics of Romidepsin in Patients with Advanced Cancer	Hematologic Malignancy, Malignant Lymphoma	Phase 1	Completed	NCT01324310
Romidepsin	Phase II Study of Gemcitabine+Romidepsin in the Relapsed/Refractory Peripheral T-cell Lymphoma Patients (FIL_GEMRO)	Peripheral T-cell Lymphoma	Phase 2	Completed	NCT01822886
Romidepsin	A Rollover Study for Patients who Participated in other Romidepsin Protocols	Lymphoma, Cancer	Phase 2	Completed	NCT01353664

Plitidepsin	Aplidin-Dexamethasone in Relapsed/Refractory Myeloma (ADMYRE)	Relapsed/Refractory Multiple Myeloma	Phase 3	Completed	NCT01102426
Plitidepsin	Multicenter Trial to Treat Patients with Relapsed/Refractory Aggressive Non-Hodgkin Lymphoma	Leukemia, Lymphoma	Phase 2	Completed	NCT00884286
Plitidepsin	Clinical Trial of Aplidin® in Patients with Primary Myelofibrosis	Myelofibrosis	Phase 2	Completed	NCT01149681
Plitidepsin	Study of Plitidepsin (Aplidin®) in Combination with Bortezomib and Dexamethasone in Patients with Multiple Myeloma	Multiple Myeloma	Phase 1	Completed	NCT02100657
Plitidepsin	Study of Plitidepsin in Combination with Sorafenib or Gemcitabine in Patients with Advanced Solid Tumors or Lymphomas	Advanced Solid Tumors, Lymphomas	Phase 1	Completed	NCT00788099
Plitidepsin	A Study of Aplidin (Plitidepsin) 3 h iv in Subjects with Relapsing or Refractory Multiple Myeloma	Multiple Myeloma	Phase 2	Completed	NCT00229203
Plitidepsin	Trial of Plitidepsin (Aplidin®) in Combination with Bortezomib and Dexamethasone in Multiple Myeloma Patients Double Refractory to Bortezomib and Lenalidomide	Multiple Myeloma	Phase 2	Terminated	NCT03117361
Plitidepsin	Clinical Study of Plitidepsin (Aplidin®) in Combination with Cytarabine in Patients with Relapsed/Refractory Leukemia	Relapsed/Refractory Leukemia	Phase 1/2	Terminated	NCT00780143
Plitidepsin (Aplidin®)	A Study of Plitidepsin in Patients with Relapsed or Refractory Angioimmunoblastic T-cell Lymphoma	Lymphoma	Phase 2	Terminated	NCT03070964
Plitidepsin (Aplidin®)	A Study of Aplidin (Plitidepsin) in Subjects with Advanced Prostate Cancer	Prostate Cancer	Phase 2	Terminated	NCT00780975
Plitidepsin (Aplidin®)	Efficacy and Safety of Plitidepsin in Patients with Advanced Unresectable or Metastatic, Relapsed/Refractory, Dedifferentiated Liposarcoma (DLPS): An Exploratory Phase II Multicenter Trial (APLIPO)	Adult Patients with Unresectable Locally Advanced or Metastatic, Relapsed/Refractory Dedifferentiated Liposarcoma	Phase 2	Terminated	NCT01876043
Plitidepsin (Aplidin®)	Proof of Concept Study to Evaluate the Safety Profile of Plitidepsin in Patients with COVID-19 (APLICOV-PC)	COVID-19	Phase 1	Active, not recruiting	NCT04382066
